# PI3Kγ in Tumour Inflammation: Bridging Immune Response and Cancer Progression—A Mini‐Review

**DOI:** 10.1111/imm.13959

**Published:** 2025-05-28

**Authors:** Anghesom Ghebremedhin, Judith A. Varner

**Affiliations:** ^1^ Moores Cancer Center University of California San Diego California USA; ^2^ Department of Pathology University of California San Diego California USA

**Keywords:** immune suppression, integrin activation, macrophages, myeloid cell, myeloid‐derived suppressor cells, PI3Kinase, PI3Kγ, polarisation, tumour microenvironment

## Abstract

Phosphatidylinositol 3‐kinase gamma (PI3Kγ), a class I PI3K family member, plays a critical role in modulating inflammation and immune responses within the tumour microenvironment. Emerging evidence suggests that PI3Kγ promotes myeloid cell trafficking and transcription, leading to tumour progression and metastasis. This review explores the multifaceted roles of PI3Kγ in tumour‐associated inflammation, highlighting its involvement in immune cell polarisation, cytokine production, and the dynamic interaction between tumour cells and the surrounding stromal environment. We also discuss the potential therapeutic implications of targeting PI3Kγ to modulate inflammation and inhibit tumour growth. Given its pivotal role in immune response and tumour progression, PI3Kγ represents a promising target for future cancer therapies to reduce inflammation‐driven tumorigenesis.

## Graphical abstract

Myeloid cell PI3Kγ can be activated by G Protein‐Coupled Receptors or Receptor Tyrosine Kinases. Once activated, PI3Kγ promotes integrin activation as well as Akt and mTOR activation, thereby promoting transcription of immune‐suppressive and pro‐angiogenic factors, such as Arginase, IL‐10, TGFβ and VEGF‐A. PI3Kγ controls immune cell recruitment, macrophage phenotype switching, inflammatory cytokine production and tumour progression.

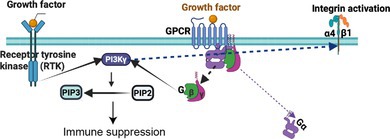

## 1 Introduction

**FIGURE 1:**
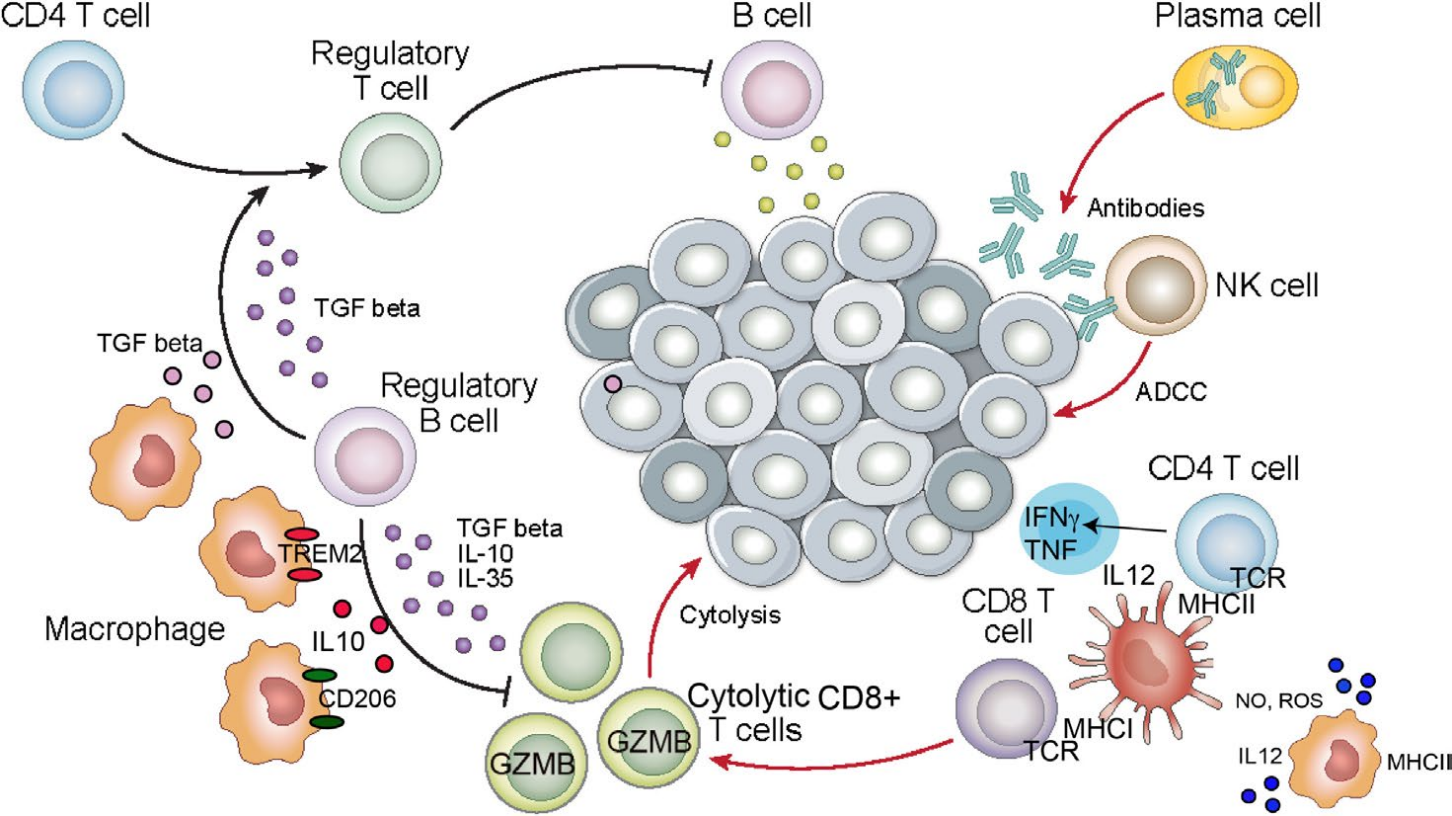
Immune cells in the tumour microenvironment. The tumour microenvironment consists of extracellular cell‐matrix components, cancer‐associated fibroblasts (CAFs), vascular and lymphatic endothelium and immune cells. Tumours can be populated by regulatory T and B cells, which secrete immune‐suppressive factors such as TGFbeta, IL10 and IL35, and immune‐suppressive macrophages, monocytes, neutrophils and dendritic cells. Tumour inhibition can be achieved when the microenvironment includes antibody‐secreting plasma cells, cytolytic T cells and NK cells, and immune‐stimulatory myeloid cells.

The tumour microenvironment (TME) can vary widely between tumour types, as well as within different regions of individual tumours. The TME consists of stromal and extracellular cell‐matrix components, and immune cells, which can include T and B lymphocytes, Natural Killer (NK) cells, plasmacytoid dendritic cells and myeloid cells. Tumour‐associated myeloid cells can include macrophages, monocytes, neutrophils, and myeloid dendritic cells [1, 2]. Tumour‐associated macrophages (TAMs), monocytes and granulocytes can inhibit T cell function and are often designated as “myeloid‐derived suppressor cells (MDSCs)”. Together these cells can comprise a large proportion of the cellular population within tumours. Tumour‐associated myeloid cells typically exhibit chronic wound‐healing, immunosuppressive phenotypes; a high myeloid cell content is generally prognostic of poor cancer outcome (Figure 1) [3, 4, 5, 6].

Monocytes and granulocytes are recruited from the earliest phases of carcinogenesis by chemokines of the CC and CXC families, such as CCL2, CCL5 and CXCL12 [7, 8, 9]. Cancer cells, TAMs, and other cells in the TME, such as carcinoma‐associated fibroblasts (CAFs), are major sources of such chemoattractants [2, 4, 5]. Once in the neoplastic environment, monocytes may differentiate into macrophages, a process that is facilitated by tumour‐derived haematopoietic growth factors, such as monocyte/macrophage colony‐stimulating factor (M‐CSF) and granulocyte‐monocyte colony‐stimulating factor (GM‐CSF) [10, 11, 12, 13, 14, 15]. Tumour‐associated macrophages (TAMs) can include pro‐inflammatory M1‐like subtypes and the immunosuppressive M2‐like subtypes. A higher ratio of M2‐like to M1‐like TAMs indicates a more immunosuppressive tumour microenvironment. Other cytokines and molecules in the tumour microenvironment (e.g., IL‐10, TGFβ‐1 and PGE) also regulate tumour‐associated myeloid cells, as they play significant roles in the functional polarisation of monocytes/macrophages into an immunosuppressive phenotype [9].

TAMs are a major source of CCL8, which, together with SIGLEC1, participates in a tumour cell–TAM positive feedback regulatory loop that leads to increased tumour cell motility. In response to CCL8, cancer cells secrete CSF1, a key survival and proliferation factor for macrophages, further amplifying the auto‐stimulatory loop. The elevated concentration of CCL8 not only supports the cancer–TAM crosstalk but also acts as a monocyte chemoattractant [16].

TAMs can be derived either from monocytes that are recruited from the circulation or from tissue resident macrophages. Resident macrophages are seeded into tissues during embryonic development by yolk sac or fetal liver progenitors, which self‐renew locally in tissues [17, 18]. In addition to TAMs, tumour‐associated neutrophils (TANs) are found in the TME [19]. Neutrophils originate in the bone marrow, and their maturation is orchestrated by GM‐CSF, G‐CSF and IL‐6. Circulating neutrophils express the chemokine receptors CXCR1 and CXCR2, and their recruitment to tumour tissues is regulated by CXCL1 and CXCL2, which are the focus of several immune therapy strategies [20]. Like TAMs, tumour‐associated neutrophils (TANs) can also be classified into N1‐like (anti‐tumorigenic) and N2‐like (pro‐tumorigenic) subtypes.

A better understanding of the molecular and cellular mechanisms involved in the recruitment and diversity of myeloid cells in cancer is essential for the development of specific therapeutic strategies targeting TAMs and TANs. The growing understanding of immunosuppressive myeloid cell subsets and their role in promoting cancer has led to interest in therapeutic targeting of these cells, either by depletion, inhibition of recruitment, or modulation of their phenotypes. Multiple cell surface receptors can promote the recruitment of monocytes and granulocytes into the tumour microenvironment as well as their polarisation towards the pro‐tumorigenic (M2‐ or N2‐like) or anti‐tumorigenic (M1‐ or N1‐like) phenotypes. The signalling protein PI3Kγ plays a central role in the recruitment of myeloid cells to the TME and in the polarisation of myeloid cells toward the immune suppressive phenotype in most tumour subtypes. In this context, we discuss the role of PI3Kγ signalling in tumour progression and its potential as a therapeutic target in cancer treatment.

## 2 PI3K Family of Lipid Kinases: Structure and Function

Phosphatidylinositol 3‐kinases (PI3K) are lipid kinases that specifically phosphorylate the hydroxyl group of the inositol ring of phosphatidylinositol (PtdIns) at the 3′ position. PI3Ks consist of a catalytic and an adaptor subunit and function as lipid kinases that convert phosphatidylinositol (4,5) bisphosphate (PIP2) to phosphatidylinositol (3,4,5) triphosphate (PIP3) on cell membranes. PIP3 then serves as a docking site for signalling proteins that contain a pleckstrin homology (PH) domain [21]. The phosphoinositide 3‐kinase (PI3K) family is composed of many evolutionarily conserved lipid kinases with critical roles in a wide array of biological processes, including cancer [22]. Signalling downstream of PI3Ks promotes many important cell activities, including proliferation, differentiation, metabolism, intracellular trafficking, and motility, via effector proteins such as protein kinase B (PKB)/AKT and mechanistic target of rapamycin (mTOR) protein kinase.

**FIGURE 2:**
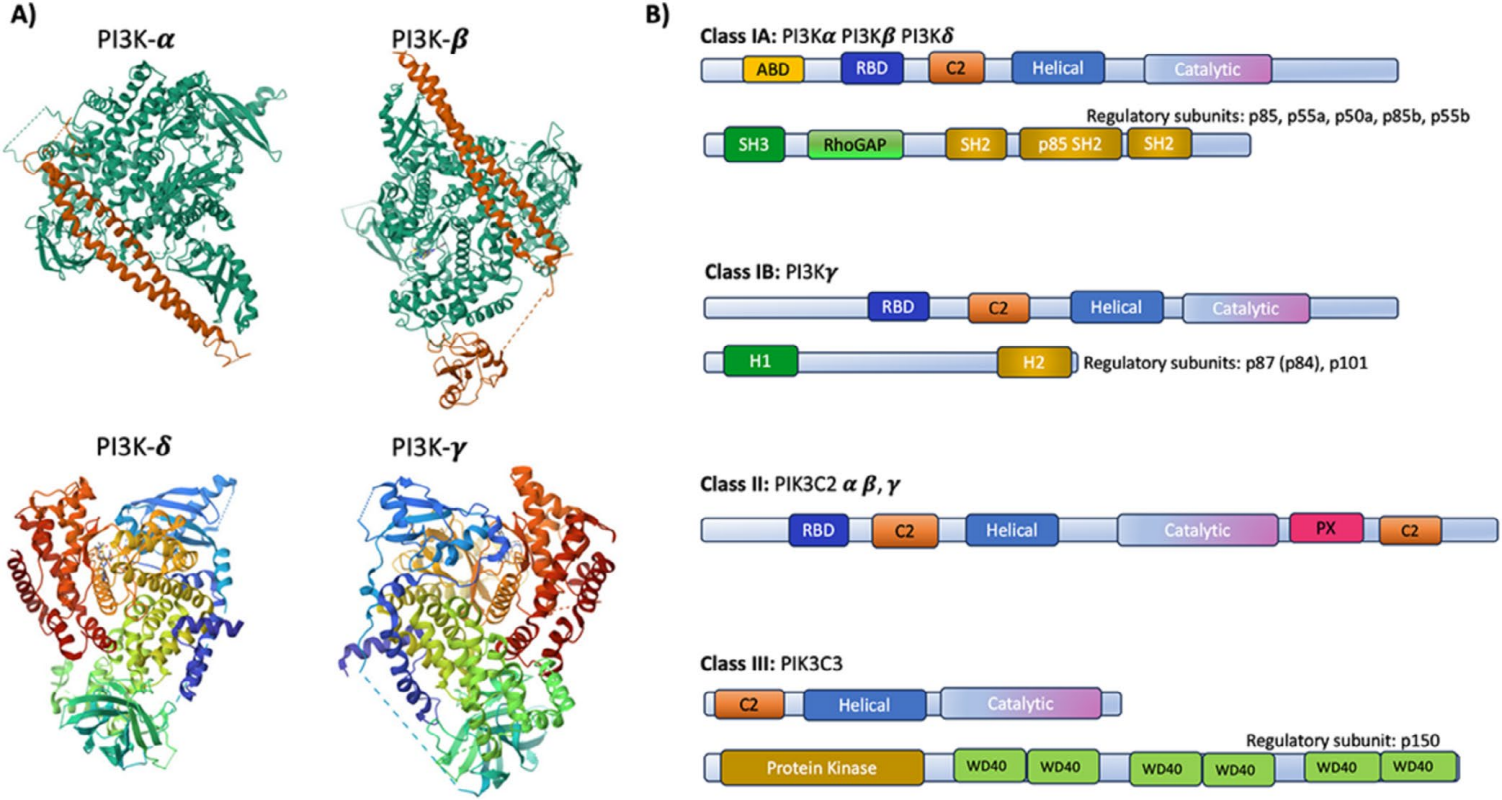
Structure of the phosphoinositide 3‐kinase (PI3K) family: (A) Crystal structures of the different Class I PI3K family isoforms p110alpha/p85alpha complex, PI3K catalytic subunit beta isoform, PI3K p110gamma catalytic domain, as well as PI 3‐kinase p110delta. (B) The PI3K family comprises three classes (I–III) that share conserved catalytic, helical, and C2 lipid‐binding domains. Class I PI3Ks are heterodimers with catalytic p110 and regulatory p85/p101 subunits, while Class II PI3Ks are monomeric with a single catalytic subunit, and Class III PI3Ks are also monomeric. Class I and II PI3Ks also contain Ras‐binding domains, and some possess accessory binding domains (ABD). Regulatory subunits direct PI3K localization and activity. Class I PI3Ks, divided into IA and IB subclasses, generate PtdIns(3,4,5)P_3_ at the plasma membrane, promoting signalling pathways involved in cell growth, survival, and migration. Class II PI3Ks produce PI(3)P and PI(3,4)P_2_, with functions in endocytosis and mitosis. Class III PI3K is primarily involved in autophagy and vesicular trafficking through PI(3)P generation.

The PI3Ks are divided into class I, class II and class III, based on their primary structure, regulation, in vitro lipid substrate specificity, and sequence homology (Figure 2) [21]. Among all types of PI3Ks, Class I PI3Ks are the most well‐studied and have been demonstrated to play important roles in cancer and immune disease, becoming attractive targets for drug development [21, 22, 23]. However, Class II and Class III members are regulators of membrane trafficking during endocytosis, endosomal recycling, and autophagy and, in these roles, may participate in tumour progression. Class I PI3Ks are subdivided into Class IA and Class IB based on their utilisation of distinct regulatory subunits. Class IA PI3Ks, which include three isoforms, PI3Kα, PI3Kβ and PI3Kδ, consist of a 110 kDa catalytic subunit and an associated 85 kDa SH2‐domain‐containing regulatory subunit (p85). The Class IB isoform PI3Kγ consists of a catalytic subunit (p110γ) and a p84/p87 or p101 regulatory subunit. PI3K regulatory subunits maintain the class I PI3K catalytic subunits in basal, inactive conformations. Upon interaction of p85‐p110 heterodimers with activated, autophosphorylated receptor tyrosine kinases, Class IA PI3Ks are recruited to the plasma membrane, where Ras‐Binding Domains (RBD) interact with Ras proteins to further activate these key kinases. In contrast, the p110γ catalytic domain can be activated by interactions of the regulatory subunit p101 with plasma membrane‐localised G protein‐coupled receptor Gβγ subunits or by interactions of p84/p87 regulatory subunits with RTKs. Further interactions with Ras are required for PI3Kγ activation.

### 2.1 The Role of Class I PI3K Isoforms in Different Cancer Subtypes

PI3K signalling is frequently altered in cancers through mutations, amplifications, or deletions in various tumour types, leading to hyperactivation of downstream signalling pathways, including the AKT/mTOR pathway. These alterations drive key hallmarks of cancer such as sustained proliferative signalling, evasion of growth suppressors, resistance to cell death, and increased metastasis. Class I PI3Ks, comprising the PI3Kα, PI3Kβ, PI3Kδ, and PI3Kγ isoforms, are the most studied class of PI3Ks in the context of tumorigenesis. These isoforms have been implicated in key aspects of cancer biology, including tumour initiation, progression, and metastasis. Among them, PI3Kα plays a particularly well‐characterised role in cancer, especially in breast cancer, where mutations in the PIK3CA gene (encoding PI3Kα) lead to constitutive activation of the pathway. This aberrant activation of PI3Kα then promotes cell survival, growth, and resistance to apoptosis, contributing to tumorigenesis and metastasis. Notably, PIK3CA mutations are present in approximately 30%–40% of breast cancers, and these mutations are associated with poor prognosis and resistance to standard therapy [24].

PI3Kβ, another isoform of Class I PI3Ks, has been shown to play a prominent role in the growth and survival of certain cancers. PI3Kβ (encoded by PIK3CB) is particularly important in tumours with PTEN loss, a common event in cancers such as prostate cancer, glioblastoma, endometrial cancer and triple‐negative breast cancer (TNBC). In PTEN‐null prostate cancer, PI3Kβ is the dominant isoform driving AKT activation and inhibition of PI3Kβ effectively reduces tumour cell proliferation in preclinical models [25]. Similarly, in glioblastoma multiforme (GBM), where PTEN mutations are frequent, PI3Kβ plays a compensatory role in sustaining downstream signalling and tumour cell survival. Selective PI3Kβ inhibition has been shown to induce apoptosis and reduce tumour growth in PTEN‐deficient glioma models [26]. Additionally, triple‐negative breast cancers, which often exhibit PTEN deficiency, show increased dependence on PI3Kβ signalling. Combination therapies targeting both PI3Kβ and mTORC1 pathways are under investigation to overcome resistance mechanisms [27].

PI3Kγ (encoded by PIK3CG), a member of the Class I PI3K family, is primarily expressed in immune cells such as neutrophils, macrophages, and, to a lesser extent, in T lymphocytes; this kinase plays a significant role in immune cell trafficking and function. Moreover, it influences both the immune response to the tumour and the tumour's ability to evade immune surveillance [28], particularly in solid tumours like pancreatic ductal adenocarcinoma (PDAC), melanoma, and lung adenocarcinoma. In PDAC, PI3Kγ mediates the recruitment and immunosuppressive function of tumour‐associated macrophages (TAMs). Genetic or pharmacologic inhibition of PI3Kγ reprograms TAMs from a tumour‐promoting (M2‐like) to a tumour‐fighting (M1‐like) phenotype, enhancing CD8+ T cell–mediated antitumor responses and synergising with checkpoint inhibitors [29, 30]. In melanoma, PI3Kγ inhibition similarly disrupts the immunosuppressive TME by blocking TAM‐mediated IL‐10 production and T cell exclusion, sensitising tumours to PD‐1 blockade [31]. Furthermore, in non‐small cell lung cancer, the PI3Kγ pathway in myeloid cells modulates inflammation and angiogenesis, contributing to a pro‐tumorigenic environment. Targeting PI3Kγ in these models reduces tumour burden and enhances responsiveness to anti‐PD‐L1 therapy [32].

PI3Kδ (encoded by the PIK3CD gene), on the other hand, is predominantly expressed in haematopoietic cells and hence has been linked to various hematologic malignancies, including chronic lymphocytic leukaemia (CLL) and lymphoma. In CLL, mutations in the PIK3CD gene lead to constitutive activation of this isoform, enhancing the survival of leukaemic cells and their resistance to therapy. Targeting PI3Kδ with selective inhibitors has shown promising results in clinical trials for B‐cell malignancies [33, 34].

### 2.2 PI3Kγ Signalling and Activation

Among the four different class I PI3K catalytic subunits, p110δ and p110γ are the two PI3Ks that are primarily expressed in cells of the immune system. p110δ binds to the p85 regulatory subunit to form PI3Kδ, and p110γ pairs with either p101 or p84 to form PI3Kγ. Both kinases are critical for healthy immune function in humans, as underscored by recently discovered primary immunodeficiency disorders that are caused by mutations in genes encoding the PI3Kδ or PI3Kγ catalytic or regulatory subunits [35, 36, 37, 38, 39, 40]. PI3Kγ is preferentially expressed in myeloid cells compared to the other class I PI3Ks [41], and its involvement in mediating myeloid cell recruitment and tumorigenesis will be explored in the sections that follow.

PI3Kγ can associate with two unique regulatory subunits, p84/p87 and p101. The Class I B isoform, PI3Kγ, is highly expressed in the myeloid lineage, including neutrophils, macrophages, and mast cells [41, 42, 43]. PI3Kγ is primarily activated by trimeric G proteins and RAS family GTPases, and it converts PIP2 to PIP3, which canonically occurs at the plasma membrane. Trimeric G proteins and RAS‐family proteins induce the recruitment of PI3Kγ to membranes, thereby facilitating allosteric changes upon lipid binding that elicit enzyme activation. GPCRs dissociate G proteins to release the Gβγ subunit that binds to the adaptor protein of PI3Kγ, enhancing p110γ lipid kinase activity [41, 42, 43]. Furthermore, PI3Kγ p84–p110γ complexes produce PIP3 functional pools and activate PI3Kγ in a RAS–GTP dependent manner [41, 43]. Once activated, PI3Kγ transfers the γ‐phosphate of ATP to phosphatidylinositol (4,5) bisphosphate (PIP2) to produce phosphatidylinositol (3,4,5) trisphosphate (PIP3), which serves as a docking site for effector proteins, such as AKT.

## 3 PI3Kγ Roles in Tumour Progression and Inflammation

Approximately 15% of cancer types arise in association with chronic inflammation, indicating a link between cancer and inflammation. The cause of inflammation varies and has been attributed to infectious agents such as *Helicobacter pylori* /gastric cancer, hepatitis C/liver cancer, and papillomavirus/cervical cancer. Chronic inflammation related to environmental toxins (asbestos, coal dust, and tobacco smoke), autoimmune disorders (Crohn's disease), and obesity can also induce cancer [44, 45, 46]. In chronically inflamed tissues as well as tumours, there is an extensive infiltration of tissues by immunosuppressive macrophages [47, 48]. A large fraction of inflammatory cells in tumours are TAMs, which express numerous factors that promote a pro‐tumorigenic microenvironment by stimulating angiogenesis, metastasis, inflammation, and immunosuppression, as well as relapse after therapy [45]. Furthermore, in patients, TAMs and MDSCs (Myeloid‐derived suppressor cells) are associated with poor survival in numerous cancer types.

Such innate immune cells are recruited to chronically inflamed tissues and tumours by diverse chemoattractants, including CXCL1, CXCL2, CCL2, CCL3, CCL4 and more chemokines and cytokines. These chemoattractants induce integrin‐mediated myeloid cell adhesion to endothelium and subsequent extravasation into tissues, in a process known as trafficking [49] in response to the activation of G protein‐coupled receptors (GPCR), receptor tyrosine kinases (RTK), and/or Toll‐like/interleukin‐1 receptors (TLR/IL1R) that initiate myeloid cell recruitment [41, 49, 50, 51, 52]. These receptors stimulate myeloid cell recruitment by activating PI3Kγ in circulating myeloid cells [41]. GPCRs activate p110γ in a Ras/p101‐dependent manner, while RTKs and TLR/IL1Rs directly activate p110γ in a Ras/p87‐dependent manner. Once activated, p110γ in turn activates integrin α4β1, causing granulocytic and monocytic cell adhesion to endothelium and invasion into tumours [41, 53].

Integrins can be activated by a process known as “inside‐out” signalling, when receptor‐mediated signal transduction results in conformational changes in integrin extracellular domains that increase their capacity to bind ligands and therefore attach to endothelium and extracellular matrices [54, 55]. Such integrin activation is required for lymphocyte and myeloid cell extravasation from the vasculature [41, 49]. Integrin activation depends on PI3Kγ‐mediated activation of Rap1 (Ras‐proximate‐1), a Ras‐like small GTP‐binding protein [53, 56, 57, 58]. Rap1 activates the effector protein, RIAM, which interacts closely with the cytoskeletal protein talin, thereby localising it near the membrane [57, 58, 59]. Talin then binds to sites in the integrin β chain cytoplasmic tails, disrupting electrostatic interactions between amino acids in the integrin α and β chain cytoplasmic tails and altering the packing of integrin transmembrane domains [60, 61, 62]. These events alter the conformation of the extracellular domains of the integrin heterodimer and increase ligand binding. In addition, paxillin‐binding to the integrin α4 cytoplasmic tail also promotes integrin activation, as disruption of the paxillin‐binding site in the integrin α4 cytoplasmic tail partially prevents talin binding and inhibits adhesion and trafficking of lymphocytes and myeloid cells [53, 63, 64].

PI3Kγ activation by a variety of inflammatory stimuli promotes integrin α4 conformational changes, cell adhesion, and myeloid cell trafficking in vivo [41, 49]. Myeloid cell trafficking to tumours depends on PI3Kγ‐mediated integrin activation, and subsequent myeloid cell recruitment and tumour inflammation depend on PLCγ, CalDAG‐GEFI and II, Rap1a, RIAM, talin, paxillin and myosin light chain kinase [65, 66]. Pharmacological or genetic blockade of p110γ suppressed recruitment of both monocytes and granulocytes and suppressed angiogenesis, tumour growth, progression and metastasis of implanted and spontaneous tumours [41]. These findings indicated that targeting the molecular mechanisms promoting the recruitment of myeloid cells during inflammation could provide significant benefit in the treatment of a wide variety of cancers.

**FIGURE 3:**
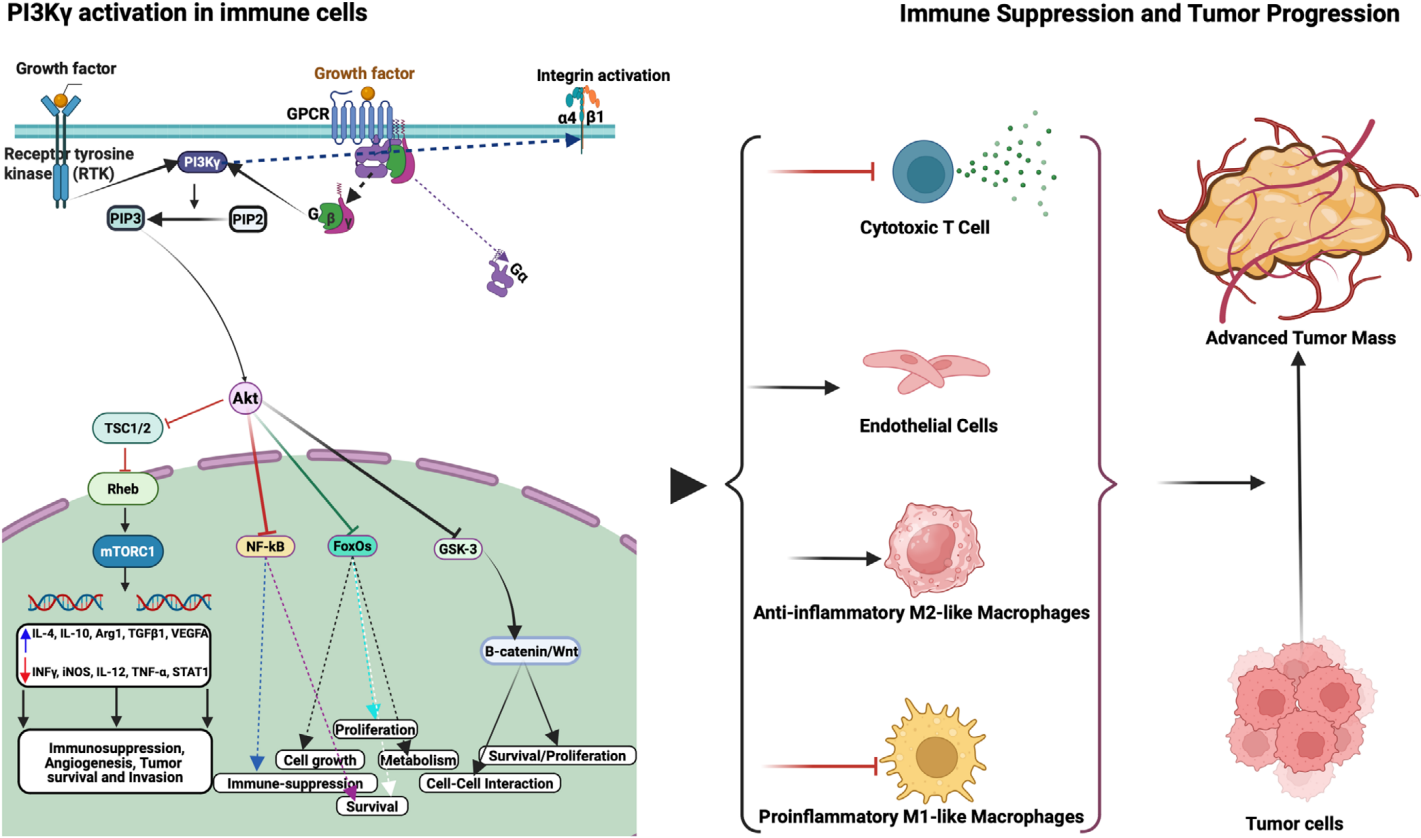
The Roles of PI3Kγ in tumour progression and inflammation. Myeloid cell PI3Kγ can be activated by G Protein‐Coupled Receptors or Receptor Tyrosine Kinases. Once activated, PI3Kγ promotes integrin activation as well as Akt and mTOR activation, thereby promoting the transcription of immune‐suppressive and pro‐angiogenic factors, such as Arginase, IL‐10, TGFβ and VEGF‐A. Through its regulation of intracellular signalling, PI3Kγ controls immune cell recruitment, macrophage phenotype switching, inflammatory cytokine production and tumour progression. The impact of PI3Kγ signalling on pathways that promote tumour progression highlights its potential as a therapeutic target in cancer and other inflammatory diseases.

In addition to regulating the recruitment of myeloid cells, PI3Kγ controls immune‐suppressive transcription in myeloid cells (Figure 3) [30]. Kaneda et al. showed that PI3Kγ inhibition promotes NFκB activation and pro‐inflammatory transcription in myeloid cells, leading to anti‐tumour T cell activation and tumour inhibition that synergises with anti‐PD1 immune checkpoint blockade [30]. Kaneda et al. also showed that PI3Kγ plays important roles in pancreatic cancer ductal adenocarcinoma (PDAC) progression, as PI3Kγ inhibition promotes survival and inhibits both desmoplasia and metastasis in the Pdx1‐Cre; LSL‐Kras^G12D^; LSL‐Tp53^R172H/+^ (KPC) mouse model of pancreatic ductal adenocarcinoma (PDAC) [30]. Targeting of PI3Kγ‐activated dendritic cells to stimulate T cell‐mediated inhibition of tumour growth, suppressed infiltration of MDSCs, and synergised with anti‐PD1 and anti‐CTLA4 therapies in preclinical models [28, 67, 68]. Further studies found that targeting PI3Kγ could disrupt a STAT3‐MYC signalling axis by remodelling the tumour‐associated microenvironment, including reducing microglia/macrophage‐associated IL11 secretion in murine glioblastoma models [69]. Moreover, PI3Kγ inhibition synergised with temozolomide in orthotopic murine glioblastoma models and with gemcitabine in a model of Kras^G12C^p53^+/−^ lung adenocarcinoma [28, 69]. These studies show that disruption of PI3Kγ signalling serves effectively as a promising cancer therapy.

## 4 Therapeutic Potential of **PI3Kγ** Inhibitors

Links between PI3K family members and oncogenesis have been established, placing the PI3K pathway as one of the most mutated or amplified pathways in tumours [26]. PI3K was discovered in the late 1980s, and the first PI3K inhibitors (Wortmannin and LY294002) were described in 1994 [29]. Recent advances have enabled breakthroughs in the development of next‐generation small molecules that inhibit PI3Kγ with improved potency and specificity. IPI‐549, an oral PI3Kγ‐selective inhibitor, exhibits an IC50 value of 0.29 nM against PI3Kγ, 17 nM against PI3Kα, 82 nM against PI3Kβ and 23 nM against PI3Kδ [67]. IPI‐549, which has 60‐fold or greater selectivity for PI3Kγ over other PI3K isoforms and other kinases, thus showed good potential for advancing to clinical trials. Additional PI3Kγ inhibitors have been developed and evaluated in phase I clinical trials, including TG100‐115 (Targegen), Duvelisib (Infinity Pharmaceuticals), AZD8154 (AstraZenica), and ZX‐4081 (Nanjing Zenshine Pharmaceuticals) [70, 71, 72, 73]. These inhibitors exhibited good safety profiles.

IPI‐549, in conjunction with T cell checkpoint blockade or chemotherapy, has been tested in phase I and II clinical trials for cancers including non‐small‐cell lung cancer, melanoma, head and neck squamous cell carcinoma, triple‐negative breast cancer, and urothelial carcinoma (NCT03961698, NCT03980041, and NCT03795610) [32, 74]. Early data from these clinical trials have shown increases in overall response rates in the PI3Kγ inhibition groups (combined with nivolumab) compared to nivolumab alone [32, 74]. IPI‐549 in combination with PD1 checkpoint blockade also elevated overall response rates in patients who were unresponsive to prior checkpoint therapies owing to low PDL1 expression [74]. Efficacy in tumours with low PDL1 expression is especially encouraging for the future therapeutic use of PI3Kγ inhibitors because tumours that express low levels of this biomarker are difficult to treat. Analysis of key biomarkers showed that PI3Kγ inhibition promoted the expression of inflammatory cytokines in blood (e.g., CXCL9/10) and in tissues.

## 5 Conclusions and Future Perspectives

Tumour tissues are populated by a plenitude of myeloid cells that are highly heterogeneous in function, localization and morphology and engage in complex bidirectional interactions with tumour cells, tumour‐infiltrating lymphocytes, as well as other stromal components. Among immune cell populations, TAMs and TANs are major drivers of cancer immune suppression and are emerging as both indicators of cancer prognosis and promising targets for therapeutic intervention. The most extensively studied myeloid targeting drugs are modulators of colony‐stimulating factor 1 receptor (CSF1R), CC‐chemokine receptor 2 (CCR2), CXC‐chemokine receptor 2 (CXCR2) and phosphoinositide 3‐kinase γ (PI3Kγ). Despite promising results from single‐arm phase I and small phase II clinical trials, phase III trials of CSF1R, CCR2 or CXCR2 inhibitors in broad patient populations have not yet shown compelling efficacy, and treatment has been associated with toxicity challenges. This suggests that, as with many cancer therapies, patient‐selection biomarkers and combination therapies will be required for the therapeutic success of myeloid targeting agents.

PI3K inhibitors have demonstrated significant potential in cancer treatment, but challenges such as off‐target effects, toxicity and resistance have limited their clinical effectiveness. The future of PI3K inhibition will likely rely on more selective inhibition, including isoform‐specific inhibitors like those targeting PI3Kγ, which is predominantly expressed in immune cells.

PI3Kγ inhibitors are expected to offer a more refined therapeutic approach, particularly in oncology and other inflammatory ailments. By specifically targeting PI3Kγ, these inhibitors aim to mitigate systemic side effects associated with pan‐PI3K inhibition while harnessing the immune‐modulatory potential of PI3Kγ inhibition. For instance, recent studies have shown that PI3Kγ inhibition can enhance anti‐tumour immunity, synergize with immune checkpoint inhibitors and potentially overcome immune evasion mechanisms [28, 41, 67, 68, 69, 75]. Additionally, the application of PI3Kγ inhibitors in inflammatory diseases such as SARS‐CoV‐2 infections, rheumatoid arthritis, and atherosclerosis shows promise, as they could selectively modulate immune responses without broad immune suppression [31, 76].

One of the key areas of future research on PI3Kγ inhibitors will be optimising combination therapies with other targeted therapies, checkpoint inhibitors, or standard chemotherapy to improve patient outcomes. The development of biomarkers to predict response, as well as strategies to overcome resistance mechanisms, will also be crucial. Personalised medicine approaches, including the use of biomarkers to guide therapy selection, will also likely improve the efficacy and safety profile of these therapies. Ultimately, the success of PI3Kγ inhibitors may rely on refining their activity to achieve maximum therapeutic efficacy with minimal adverse effects, paving the way for their integration into more comprehensive therapeutic strategies for cancer and inflammatory diseases.

## Author Contributions

Conceptualisation: J.A.V. and A.G. Writing – original draft preparation: A.G. Writing – review and editing: J.A.V. and A.G. Visualisation: J.A.V. and A.G. All authors have read and agreed to the published version of the manuscript.

## Conflicts of Interest

J.A.V. was a consultant for Infinity Pharmaceuticals from 2020 to 2023. J.A.V. is a shareholder in Impact Biosciences and AlphaBeta Therapeutics. A.G. declares no conflicts of interest.

## Data Availability

Data sharing not applicable to this article as no datasets were generated or analysed during the current study.
